# Bioactive calcium phosphate materials and applications in bone regeneration

**DOI:** 10.1186/s40824-018-0149-3

**Published:** 2019-01-14

**Authors:** Jiwoon Jeong, Jung Hun Kim, Jung Hee Shim, Nathaniel S. Hwang, Chan Yeong Heo

**Affiliations:** 1 Interdisciplinary Program in Bioengineering, Seoul National University, Seoul, 152-742 Republic of Korea; 2 Department of Plastic and Reconstructive Surgery, College of Medicine, Seoul National University, Seoul, Republic of Korea; 3 Department of Plastic and Reconstructive Surgery, Seoul National University Bundang Hospital, Seongnam, Republic of Korea; 4 School of Chemical and Biological Engineering, Institute of Chemical Processes, Seoul National University, 1 Gwanak-ro, Gwanak-gu, Seoul, 151-742 Republic of Korea; 5 N-Bio/BioMAX Institute, Seoul National University, Seoul, 152-742 Republic of Korea

**Keywords:** Calcium phosphate, Bone regeneration, Hydroxyapatite, Tricalcium phosphate, Whitlockite, Bone regenerative application

## Abstract

**Background:**

Bone regeneration involves various complex biological processes. Many experiments have been performed using biomaterials in vivo and in vitro to promote and understand bone regeneration. Among the many biomaterials, calcium phosphates which exist in the natural bone have been conducted a number of studies because of its bone regenerative property. It can be directly contributed to bone regeneration process or assist in the use of other biomaterials. Therefore, it is widely used in many applications and has been continuously studied.

**Mainbody:**

Calcium phosphate has been widely used in bone regeneration applications because it shows osteoconductive and in some cases osteoinductive features. The release of calcium and phosphorus ions regulates the activation of osteoblasts and osteoclasts to facilitate bone regeneration. The control of surface properties and porosity of calcium phosphate affects cell/protein adhesion and growth and regulates bone mineral formation. Properties affecting bioactivity vary depending on the types of calcium phosphates such as HAP, TCP and can be utilized in various applications because of differences in ion release, solubility, stability, and mechanical strength. In order to make use of these properties, different calcium phosphates have been used together or mixed with other materials to complement their disadvantages and to highlight their advantages. Calcium phosphate has been utilized to improve bone regeneration in ways such as increasing osteoconductivity for bone ingrowth, enhancing osteoinductivity for bone mineralization with ion release control, and encapsulating drugs or growth factors.

**Conclusion:**

Calcium phosphate has been used for bone regeneration in various forms such as coating, cement and scaffold based on its unique bioactive properties and bone regeneration effectiveness. Additionally, several studies have been actively carried out to improve the efficacy of calcium phosphate in combination with various healing agents. By summarizing the properties of calcium phosphate and its research direction, we hope that calcium phosphate can contribute to the clinical treatment approach for bone defect and disease.

## Background

Bone regeneration is intertwined with complex physiological processes by various materials and conditions [1], and interactions between environment conditions and substrates lead to a balance between osteoclasts and osteoblasts [2]. Bone regeneration has been extensively investigated in the clinical field using biomaterials. It is clinically complex and involves many biological processes. Numerous studies on areas such as the relationship between osteoclasts and osteoblasts, osteogenic differentiation, stimulation effects of bone, cell growth, signaling pathways, and bone growth factors have been conducted in vitro and in vivo [2–4].

Biomaterials should be biologically stable and biocompatible in the body and elicit no immune response [5]. Materials used in clinical applications include polymers, metals, and carbon-based ceramics [6]. However, these materials show disadvantages such as poor mechanical properties, low biocompatibility, and poor adhesion to human tissues [7]. To overcome these issues, calcium phosphate-based ceramics, which are abundant in native human bone, have begun to emerge as suitable biomaterials [8]. Calcium phosphates have been reported to possess osteoconductive and osteoinductive characteristics, and they aid in the osteogenic differentiation of mesenchymal stem cells [9, 10]. Therefore, many studies on the use of calcium phosphates for bone regeneration have been conducted, and applications in bone regeneration are actively being developed. In this review, we will summarize bone regenerative strategies using calcium phosphate by examining the bioactive properties and bone regenerative applications of calcium phosphate.

## Bioactivity of calcium phosphate

**Fig. 1:**
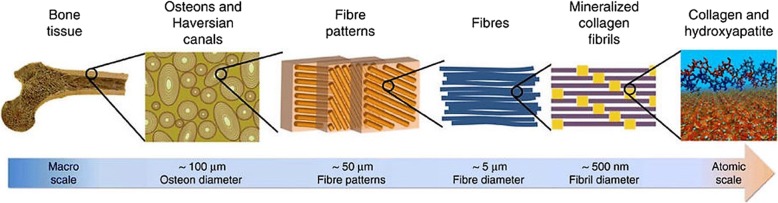
Hierarchical structure of bone ranging from macroscale skeleton to nanoscale collagen and HAP [171]

Calcium phosphates are minerals composed of calcium cations and phosphate anions. They are known as the major inorganic material in approximately 60% of all native human bones (Table 1). The existence of calcium phosphates in bones was first discovered in 1769, and in the 1800s, calcium phosphates that exist in bones were subdivided into different categories (Fig. 1) [11, 12]. Since the 1900s, synthetic calcium phosphates have been actively studied for clinical use [13–15]. Thereafter, bone regenerative applications such as bone cements, scaffolds, implants, and coating techniques using calcium phosphates have emerged, and some have been commercialized [16–18]. Similar to these, the characteristics of calcium phosphates have been studied for bone regenerative applications.

**Table 1:** Typical compositional values of the inorganic phase of adult human calcified tissues [182]

Composition	Enamel	Dentin	Bone	Hydroxyapatite
Calcium [wt.%]	36.5	35.1	34.8	39.6
Phosphorus [wt.%]	17.7	16.9	15.2	18.5
Ca/P (molar ratio)	1.63	1.61	1.71	1.67
Sodium [wt.%]	0.5	0.6	0.9	–
Magnesium [wt.%]	0.44	1.26	0.72	–
Potassium [wt.%]	0.08	0.05	0.03	–
Carbonate [wt.%]	3.5	5.6	7.4	–
Fluoride [wt.%]	0.01	0.06	0.03	–
Chloride [wt.%]	0.30	0.01	0.13	–
Pyrophosphate [wt.%]	0.022	0.10	0.07	–
Total inorganic [wt.%]	97	70	65	100
Total organic [wt.%]	1.5	20	25	–
Water [wt.%]	1.5	10	10	–
Ignition products (800 °C)	β-TCP + HAP	β-TCP + HAP	HAP + CaO	HAP

Every implantable material must be biocompatible, meaning that inflammation or foreign body response should not occur in the living system and tissue. Calcium phosphates were discovered to be biocompatible because they can be dissolved in body fluids and are present in large amounts in solid forms [19].

The properties of calcium phosphates affect bioactivity, such as adhesion, proliferation, and new bone formation in osteoblasts. To exhibit these bioactive features, degradation and ion release in calcium phosphates are important [19]. These phenomena increase the local concentration of calcium and phosphate ions and stimulate the formation of bone minerals on the surface of calcium phosphates. They also affect the expression of osteoblastic differentiation markers such as COL1, ALP, BMPs, OPN, OCN, BSP, ON, and RunX2 [20–24]. Calcium phosphates play important roles in cell adhesion and tissue formation by affecting the adsorption of extracellular matrix proteins on the surface [25, 26]. Their properties also influence bone regeneration by affecting newly formed bone minerals [27].

First, calcium ions affect cells and living systems in several ways. Calcium is one of the ions that form the bone matrix, and it exists mostly in the form of calcium phosphates in bone tissues [28]. These calcium ions cause bone formation and maturation through calcification. In addition, calcium ions affect bone regeneration through cellular signaling. Calcium stimulates mature bone cells through the formation of nitric oxide and induces bone growth precursor cells for bone tissue regeneration [29, 30]. Calcium ions also stimulate the osteoblastic bone synthesis pathway by activating ERK1/2 [31] and increase the life span of osteoblasts by activating the PI3K/Akt pathways [32]. Furthermore, calcium ions regulate the formation and the resorptive functions of osteoclasts [33, 34].

Phosphorus ions are present in the human body in large amounts. They are involved in a variety of substances such as proteins, nucleic acid, and adenosine triphosphate, and they affect physiological processes [35, 36]. Over 80% of phosphorous ions are present in bone in the form of calcium phosphates along with calcium ions. Phosphorous mainly exists in the form of phosphate (PO_4_^3−^), which has great influence on tissue formation and growth [35]. Phosphate regulates the differentiation and growth of osteoblasts and the osteoblastic lineage via the IGF-1 and ERK1/2 pathways, and increases the expression of BMPs [37, 38]. In addition, phosphate has a negative feedback interaction between the RANK-ligand and its receptor signaling and regulates the ratio of RANK-ligand:OPG to inhibit osteoclast differentiation and bone resorption [39, 40].

The osteoinductive and osteoconductive features of calcium phosphates are also important for bone regeneration. Osteoinduction is the ability to induce progenitor cells to differentiate into osteoblastic lineages [41, 42], whereas osteoconduction is the ability of bone growth on the surface of materials [43]. Osteoinduction and osteoconduction support cell adhesion and proliferation [41–43]. Cell adhesion is strongly influenced by the ability to adsorb extracellular matrix proteins. It is influenced by the surface characteristics of calcium phosphates, such as surface roughness, crystallinity, solubility, phase content, porosity, and surface energy [42].

Osteoconduction and osteoinduction depend on several factors. (Some studies suggested that calcium phosphates are osteoinductive even in the absence of supplements [42].) For example, surface chemistry and surface charge affect protein adsorption, and osteoblastic differentiation occurs via the interaction between cells and the extracellular matrix. Surface morphology can also exert these effects [42].

The role of the surface roughness of calcium phosphate is determined by the grain size and particle size of the calcium phosphate crystal structure. The roughness affects protein adhesion on the calcium phosphate surface. In general, protein adhesion improves at a roughness of less than 100 nm [44, 45]. Surface roughness also has an effect on cell adhesion [46].

The porosity of calcium phosphate also has an effect on bioactivity. The increase in porosity improves contact with body fluids on the surface area. Thus, dissolution rate is enhanced [19] and the presence of pores on the surface affects protein adsorption. It has been shown that protein adsorption is enhanced when the pore size of calcium phosphate was 20–500 μm [47–49]. This effect was also observed with an increase in the number of pores. Additional, pore size impacts bone ingrowth and angiogenesis [50, 51]. At a pore size of approximately 50 μm or greater, ingrowth of blood vessels and bones was possible [52, 53]. Pore sizes of greater than 100 μm affect the mechanical strength and shape of calcium phosphate [54]. Because of the existence of pores, calcium phosphate exhibits mechanical properties such as high brittleness, low impact resistance, and low tensile stress [41]. However, its compressive strength is better than that of natural human bone, and it is used in non-load bearing implants, defect filling, and coating methods.

Hydrophilicity is a critical factor in osteogenesis regulation. Hydrophilic surfaces are essential for cell adsorption and increases fibroblastic cell response [55]. They increase the maturation and differentiation of bone cells as well as osteointegration, and they also affect cellular reactions [56, 57]. Moreover, surface hydrophilicity increases the adhesion and proliferation of osteoblasts [58, 59].

The dissolution process of calcium phosphates is affected by surface area per unit volume, fluid convection, acidity, and temperature [19, 41]. This determines the stability and solubility of calcium phosphates and generally, solubility is inversely proportional to the ratio of Ca/P ions, purity, crystal size, and surface area. Stable and low-solubility calcium phosphates show low ion exchange with their surroundings and slow recrystallization rate on the surface, thus determining protein concentration and conformation by electrostatic interaction at the charged site. On the other hand, calcium phosphates with high solubility easily change the local pH and ion concentration so that protein adhesion is affected. Protein adhesion causes cell adhesion and determines the effectiveness of bone regeneration [60–62].

## Types of calcium phosphates

**Fig. 2:**
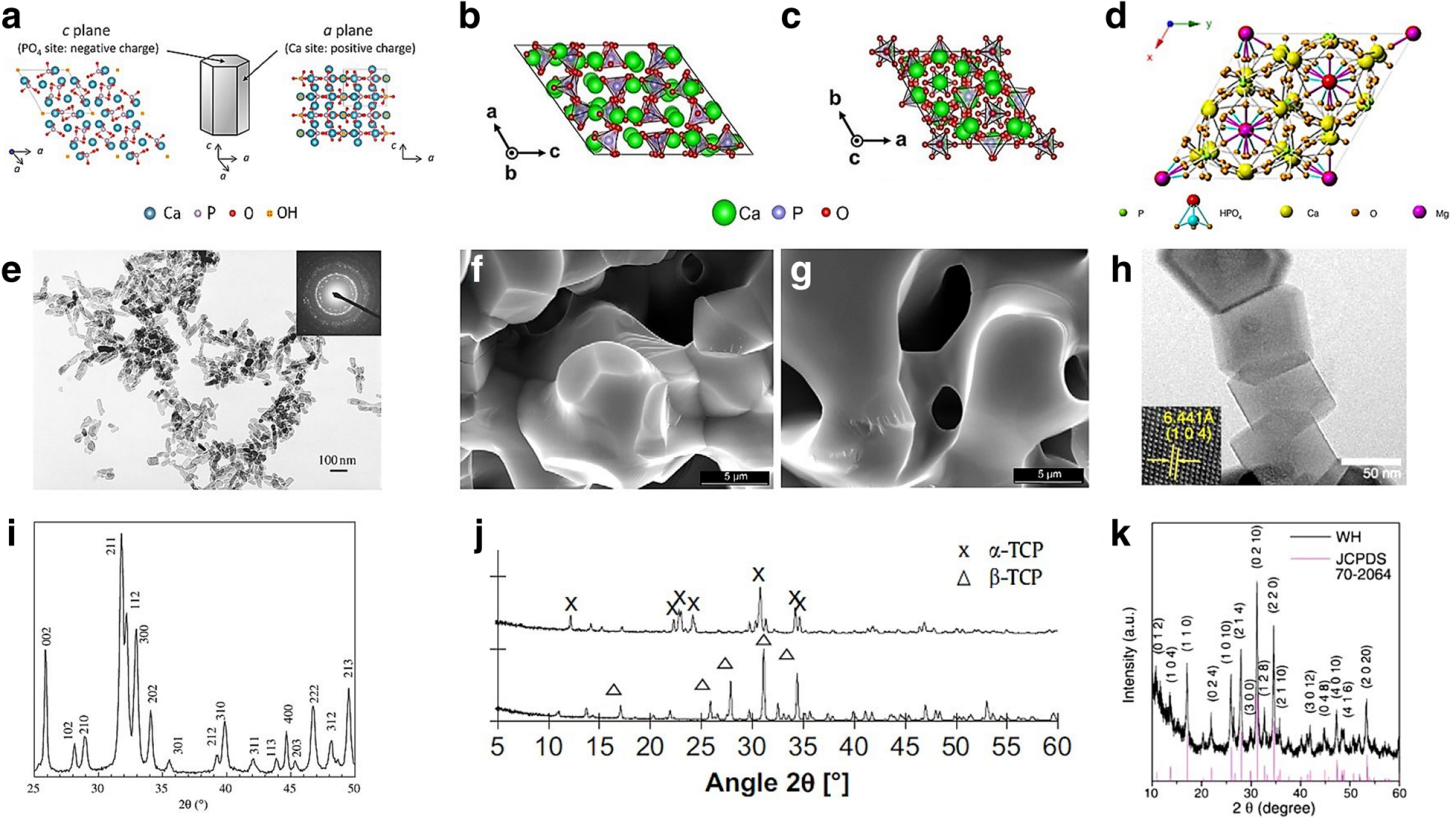
Schematic illustration of the crystal structure of (**a**) HAP [172], (**b**) α-TCP, (c) β-TCP [173], and (**d**) WH [114]. Copyright 2013 American Chemical Society. TEM and SEM images of (**e**) HAP [174], (**f**) α-TCP, (**g**) β-TCP [175], and (**h**) WH [117]. XRD data of (**i**) HAP [174], (**j**) α-TCP and β-TCP [175], and (**k**) WH [117]

As mentioned above, the osteoconductivity and osteoinductivity of calcium phosphate comes from its physical/chemical characteristics. Therefore, it is important to control these characteristics and choose the calcium phosphates with properties that are appropriate for specific applications. Calcium phosphates with bioactive features in many crystalline phases have been studied (Fig. 2).

### Hydroxyapatite

Hydroxyapatite (HAP) has been widely used in bone regeneration. It is a naturally occurring form of calcium phosphate that constitutes the largest amount of inorganic components in human bones [63]. The chemical formula of HAP is Ca_10_(PO_4_)_6_(OH)_2_ with a Ca/P ratio of 1.67 [52, 64]. HAP is naturally formed and can be collected, but various ions and vacancies form defective structures. Therefore, HAP used in actual research or clinical applications is obtained by synthesis in aqueous solution systems [65]. Stoichiometric structures can have both monoclinic and hexagonal phases, but in biological environments, they take on a hexagonal phase, which is more stable structure [66, 67]. HAP is the most stable calcium phosphate with low solubility in physiological environments defined by temperature, pH, body fluids, etc. [68, 69] and the surface of HAP can act as a nucleating site for bone minerals in body fluids [42, 70]. In addition, HAP does not cause inflammatory reactions when applied clinically [71].

HAP is known to be osteoconductive but not osteoinductive [42, 72]. Therefore, ions such as fluoride, chloride, and carbonate ions are substituted as needed [73]. For example, the use of fluoride as an anionic substitution increased the stability and the use of magnesium as a cationic substitution increased the biological effect [42]. Studies have been conducted to utilize the biocompatible characteristics of HAP, showing that in vivo bone regeneration was improved with enhancing the differentiation or promoting the proliferation of mesenchymal stem cells by increased adhesion of osteoblasts [74, 75].

Research on the clinical applications of HAP in bone regeneration began in the mid-1980s. It has been used in implant coatings [76, 77] and graft materials [78, 79], and synthetic HAP has been studied in bone regenerative applications such as granules, cements, and pastes [80, 81]. Though HAP has been investigated for clinical applications, it has not been used in cases where high load is applied because of its unique hard and brittle properties, and it has been used mainly as coatings [66, 82]. For example, coatings on the surface of metallic implants have been prepared to improve osteoblast activity [83] or to increase the contact area of bone implants [84]. In this way, HAP coatings improved the biological fixation, biocompatibility, and bioactivity of implants [85]. In addition, deposition methods such as spraying, sputtering, pulsed laser deposition, and sol-gel techniques have been attempted, and several reports have been published whereby bone formation was promoted by increasing cellular response [86–88]. Furthermore, studies on bone regenerative applications have been carried out by mixing HAP with soft materials such as polymers to complement the drawbacks. Studies are underway to control the porosity, mechanical strength, bioactivity, and ease of use, mainly using synthetic scaffolds [89–91].

### Tricalcium phosphate

Tricalcium phosphate (TCP; Ca_3_(PO_4_)_2_), one of the most studied calcium phosphates along with HAP, is a calcium phosphate with a Ca/P ratio of 1.5 and is divided into the α-phase and β-phase. α-TCP has the crystal structure of a monoclinic space group and β-TCP has the crystal structure of a rhombohedral space group [92, 93]. α-TCP can be formed at 1125 °C or higher, and β-TCP is formed at a temperature of 900–1100 °C [94, 95]. β-TCP has a more stable structure and higher biodegradation rate than those of α-TCP. Therefore, β-TCP is generally used in bone regeneration [95]. β-TCP is less stable than HAP but has a faster degradation rate and higher solubility. In addition, it has a high resorption rate and is widely used to increase biocompatibility [95, 96]. β-TCP promotes the proliferation of osteoprecursor cells such as osteoblasts and bone marrow stromal cells [97, 98]. These properties are due to the excellent biomineralization and cell adhesion by the nanoporous structure of β-TCP [99]. The characteristics of β-TCP have been actively studied for bone regeneration purposes, and β-TCP has been widely used in bone cements and bone substitution [100, 101].

In order to simultaneously utilize the characteristics of TCP and HAP, biphasic materials have been developed. Biphasic or multiphasic calcium phosphates exist in a form that is not separated because each component is homogeneously and intimately mixed at the submicron level [102]. The biphasic form of calcium phosphates was first prepared in 1986 as a mixture of HAP and β-TCP [103]. These biphasic calcium phosphates generally combine two more incompatible calcium phosphates, such as the more stable HAP and the more soluble TCP, and they have bene evaluated mainly in terms of bioactivity, bioresorbability, and osteoinductivity [104, 105]. Biphasic calcium phosphates have been used and studied as bone grafts, bone substitute materials, and dental materials [102, 106]. The mixture of HAP and β-TCP to stimulate the osteogenic differentiation of mesenchymal stem cells, increase cell adhesion, attach growth factors, and enhance mechanical properties has been actively carried out [107–109]. Ramay et al. [110] constructed a biodegradable porous nanocomposite scaffold containing a β-TCP matrix and HAP nanofibers. β-TCP/HAP scaffolds have been fabricated through gel-polymer methods and are expected to provide enhanced mechanical properties in load-bearing bone tissue engineering. The biphasic calcium phosphate scaffolds were found to have microporous structures that influenced cell growth and vascularization.

### Whitlockite

Whitlockite (WH) is a calcium phosphate-based ceramic that contains a magnesium ion and has the chemical formula Ca_9_Mg(HPO_4_) (PO_4_)_6_ [111, 112]. WH is the second most abundant mineral in human bone, occupying approximately 25–35 wt% of the inorganic portion of human bone [112, 113]. The Ca/P ratio of WH is 1.43 and it has the crystal structure of the rhombohedral space group [112, 113]. WH has high stability at acidic conditions (pH < 4.2) [114, 115] and has a negatively charged surface [116]. Compared to HAP, WH showed mechanically higher compressive strength [117]. Its solubility was higher in physiological condition and higher amount of ions could be released continuously [116].

WH has been difficult to synthesize and thus, research on WH has not progressed well. However, as a result of recent advances, it has been possible to synthesize WH easily in low-temperature conditions. It has been reported that WH is formed when Mg ions are present in acidic solutions containing calcium phosphate [118]. In addition, in vivo formation of WH occurs under acidic conditions via the release of acidic molecules when osteoclasts resorb old bone [119, 120]. Jang et al. [114] established a method for the stable formation of WH, making it easy to obtain high-purity WH without any harmful byproducts. WH analysis showed a rhombohedral shape and WH nanoparticles with a diameter of 50 nm were obtained. WH induced higher expression of osteogenic genes than did HAP and β-TCP [117]. Moreover, in vivo bone regeneration of a rat calvarial defect model with composite hydrogel showed that WH promoted growth and osteogenic activity better than HAP did [116]. These results suggested that the continuous release of magnesium and phosphate ions promoted bone growth by controlling osteogenic differentiation. Especially, magnesium ions seemed to increase bone formation because they play a role in decreasing the activity of osteoclasts [121]. It has recently been shown that osteogenic activity was increased when WH and HAP coexisted at a ratio of approximately 1:3, a similar ratio to that in native human bone [122]. These results suggested that the roles and formation mechanisms of WH in native bone need to be studied. The high osteogenic activity of WH and its role in native bone are expected to contribute to future research on calcium phosphate materials.

In addition, octacalcium phosphate (OCP), which is present in human teeth [123, 124], has a triclinic crystal structure [125] and is considered to play a role in the initial phase of HAP formation in bone mineral formation [126, 127]. OCP plays a role as a precursor of bone mineralization [128] and showed high biocompatibility [129, 130]. Thus, it has been extensively studied in bone implantation and coating [131, 132]. The amorphous form of calcium phosphate [133] has been utilized in clinical applications where certain functions are performed through ion substitution and the use of various impurities [134, 135]. Similarly, several types of calcium phosphate-based materials have been studied and utilized.

Although the bioactive properties of calcium phosphate have been studied and used for bone regeneration, there are some drawbacks such as mechanical disadvantages in clinical applications. Therefore, research has been carried out to utilize calcium phosphate as composite materials with other materials.

## Applications of calcium phosphate

**Fig. 3:**
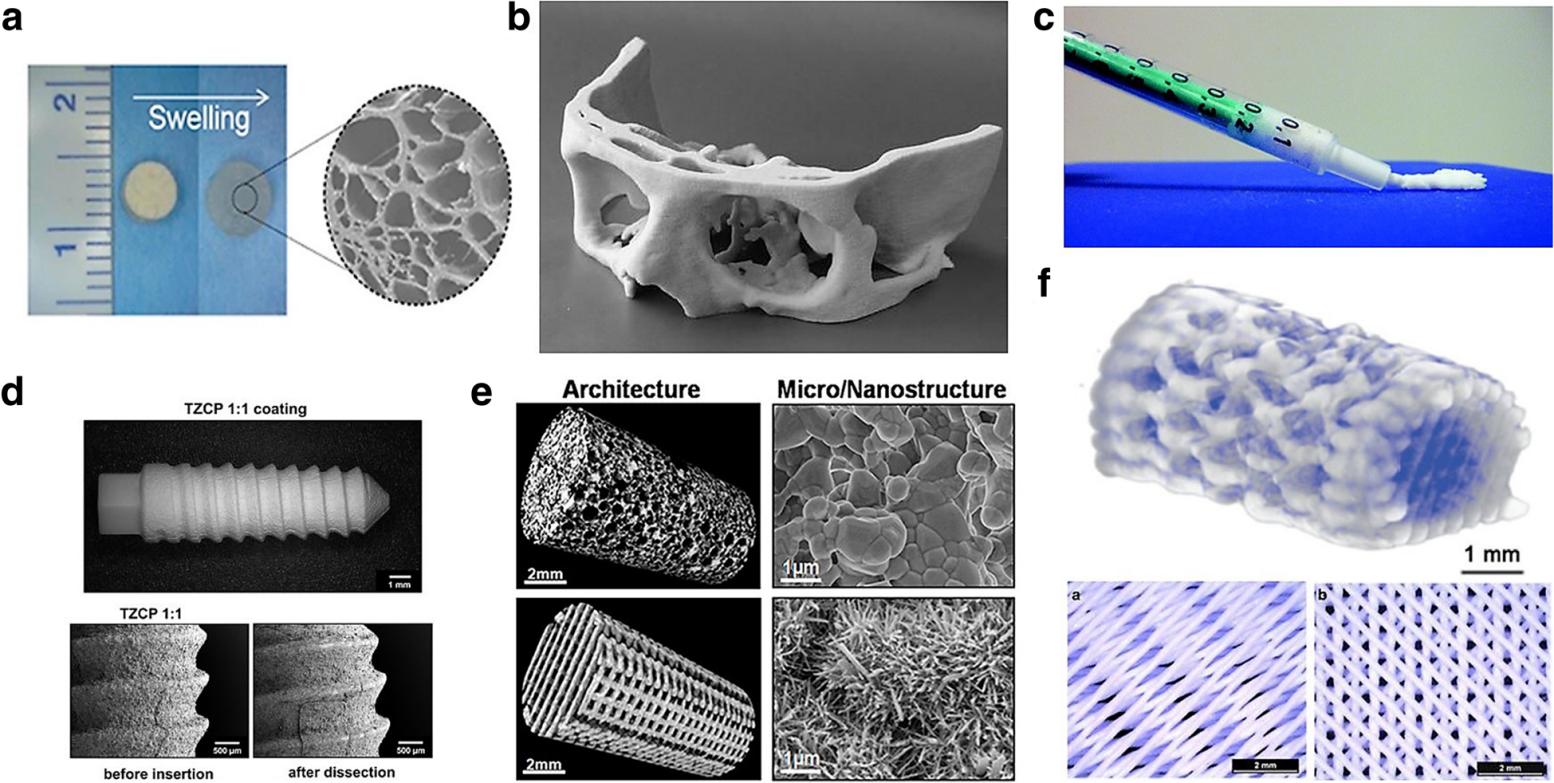
Calcium phosphate based applications. (**a**) WH incorporated hydrogel scaffold [116, 176]. (**b**) Cranial segment made of tetracalcium phosphate and β-TCP [177]. (**c**) The injectable paste included calcium phosphate nanoparticles [178]. (**d**) Mixed zirconia calcium phosphate deposited on dental implant [179]. (**e**) 3D printed calcium-deficient HAP scaffolds [180]. (**f**) 3D printed calcium phosphate cement [181]

Although calcium phosphate has been widely used for bone treatment as a raw material itself, many studies have been made using processed calcium phosphate applications for better utilization. It is used as coating materials for improving bioactivity of bone implants. And also, it is used as composites with biomaterials to alter mechanical properties, control biodegradability, and encapsulate drugs (Fig. 3).

### Coatings

Calcium phosphate coatings can be applied to various materials to enhance bioactivity. Coating of calcium phosphate is mainly performed using sol-gel and electrodeposition methods [136, 137]. Research on calcium phosphate coatings is mainly conducted for metal implant applications, aiming to prevent implant corrosion and increase bioactivity [138, 139]. Xu et al. [140] investigated porous and net-like calcium phosphate (CaHPO_4_·2H_2_O) layers coated on a magnesium alloy surface. This coating technology increased bioactivity, cytocompatibility, osteoconductivity, and osteogenesis. In vivo studies were conducted to compare this surface to that of conventional magnesium alloys. Experimental results showed that calcium phosphate-coated Mg alloy had significantly improved surface bioactivity. In the osteogenesis process, statistical differences in the expression of bone growth factor BMP-2 and TGF-β1 were observed compared to that on uncoated Mg alloys, resulting in more compact and uniform osteoid tissues.

In addition, studies on calcium phosphate coatings have resulted in improved surface reactivity and enhanced cell adhesion [141, 142]. Nguyen et al. [143] assessed the effectiveness of HAP surface coating for enhancing osteoconductivity in bone tissue engineering. They used Ti-6Al-4 V alloys with porous surfaces that were biocompatible in the human body. On top of this, a thin HAP surface was formed using a sol-gel coating technique to improve post-implantation bone ingrowth and osteoconductivity. HAP was coated on the porous surface of cylindrical implants. Using this alloy, in vivo testing of rabbit bone was carried out, and osteoconductivity was enhanced by increasing preferential protein adsorption.

Many studies have been conducted to encapsulate anti-bacterial agents and growth factors to enhance their effectiveness [144, 145]. To reduce infection and improve cell-material interaction and antimicrobial activity, AgNO_3_ and TCP were coated using the laser-engineered net shaping method on the surface of Ti metal by Roy et al. [146] Cytotoxicity assays were performed on human osteoblasts and bacterial adhesion was evaluated to assess bactericidal activity. The optimally controlled Ag-TCP-coated Ti showed a significant decrease in bacterial colonies.

### Cements

Calcium phosphate cements are used to fill and heal bone defects. Cements are mainly incorporated with polymers such as alginate, chitin, chitosan, cellulose, gelatin, collagen, and synthetic polymers such as polyethylene glycol (PEG), poly (lactic-co-glycolic acid) (PLGA), polycaprolactone (PCL), and poly (L-lactic acid) (PLLA) [147]. As a composite of these polymers, calcium phosphate cements were able to control properties such as injectability, porosity, mechanical properties, and degradation rate [147]. Hesaraki et al. [148] looked at calcium phosphate cement with improved injectability and flow for use in the urethra in vesicoureteral reflux disease and minimally invasive surgery for bone defect repair. β-TCP pastes were mixed with hyaluronic acid or PEG to make calcium phosphate cement. The enhanced viscosity and thixotropy of the calcium phosphate cement were investigated and the effect on injectability was reported.

There are some problems of calcium phosphate cements such as the difference between bone regeneration rate and degradation rate, limit of ingrowth due to pore size, lack of mechanical strength, and inflammatory reaction of synthetic polymers. Efforts are continuously being made to overcome these problems [149, 150].

Much effort has been devoted to control pore size and improve mechanical strength [151], improve degradation rate by adjusting contact with body fluid [152], add materials to improve mechanical strength [153], and minimize foreign body response by using natural polymers [154, 155]. Studies are also conducted to increase the effectiveness of cements by encapsulating drugs and growth factors [156, 157]. PLGA and calcium phosphate complex compound cements prepared for sustained delivery of recombinant human bone morphogenetic protein-2 (rhBMP-2) were investigated by Ruhe et al. [158] In this study, the rhBMP-2 release effect was measured at different pH and nanostructure conditions, suggesting that this cement can be used for bone regeneration at ectopic or orthotopic sites. Ohura et al. prepared a mixed cement of monocalcium phosphate monohydrate (MCPM) and β-TCP as another effective carrier of rhBMP-2. rhBMP-2-transplanted β-TCP-MCPM showed good effect on bone regeneration as a carrier of rhBMP-2 with suitably controlled concentration.

### Scaffolds

Calcium phosphate has been used in combination with scaffolds. Calcium phosphate scaffolds provide stable properties and allow the control of porosity and biocompatibility. The pore size of the scaffold improves revascularization and bone remodeling, enabling the ingrowth of cells and proteins and enhancing biocompatibility, making them suitable for implant use [89, 159, 160]. A variety of materials such as collagen, gelatin, PCL, PLGA, and PLLA can be used as scaffolding materials [89, 161–163]. Studies have been actively conducted to improve the bioactivity based on the characteristics and functions of various substances by enhancing the mechanical properties [164, 165], cell proliferation, and osteogenic differentiation [163, 166]. Zhao et al. [167] selected hydrogel scaffolds to improve bone regeneration. Calcium phosphates consisting of tetracalcium phosphate and dicalcium phosphate anhydrate were combined with alginate hydrogel microbeads encapsulating human umbilical cord mesenchymal stem cells to compensate for the lack of mechanical strength in the hydrogel for load-bearing. This combination could solve the difficulty in seeding cells deep within the scaffold and the inability of injection in minimally invasive surgeries. This alginate hydrogel scaffold was injectable and showed increased mechanical properties than those of conventional hydrogels.

Drugs and growth factors have been encapsulated within scaffolds [168, 169]. Koempel et al. [170] demonstrated that the integration of HAP in host bone can be promoted by attaching rhBMP-2 to macroporous ceramic HAP scaffolds. Scaffolds were implanted in rabbit calvarial defect models and after four weeks, the degree of bone formation was observed. rhBMP-2-loaded implants showed more effective bone formation. In addition, rhBMP-2 was shown to enhance osteointegration, allowing HAP scaffolds to be held in place. Therefore, it was confirmed that BMP loaded on macroporous calcium phosphate scaffolds promoted new bone formation, prevented displacement, minimized host bone resorption, and decreased the incidence of infection and extrusion.

## Summary

In summary, osteoconductive and osteoinductive features of calcium phosphate affect cell adhesion, proliferation, and new bone formation. Bioactivity can be altered and controlled by ion release and physical property of calcium phosphate on it. The ion release affects osteogenic cells, tissues, physiological processes and pathways. And then the physical property affects protein/cell absorption, promotes osteoblastic differentiation and osteointegration. Bioactive characteristics are different depending on the type of calcium phosphate such as HAP, TCP, and WH. These different bioactive characteristics are caused by the differences in Ca/P ratio, crystal structure, stability, and solubility. As mentioned above, calcium phosphates are often used with other biomaterials to control and improve their properties. Various applications have been investigated, such as coating techniques, bone cements, and composite scaffolds that have been exploited to actively utilize the bioactive features of calcium phosphate in bone regeneration.

## Abbreviations

Akt: Protein kinase B
ALP: Alkaline phosphatase
BMP: Bone-morphogenetic protein
BSP: Bone sialoprotein
COL1: Collagen type 1
ERK: Extracellular signal-regulated kinase
HAP: Hydroxyapatite
IGF: Insulin-like growth factor
MCPM: Monocalcium phosphate monohydrate
OCN: Osteocalcin
OCP: Octacalcium phosphate
ON: Osteonectin
OPG: Osteoprotegerin
OPN: Osteopontin
PCL: Polycaprolactone
PEG: Polyethylene glycol
PI3K: Phosphatidylinositol-3-kinase
PLGA: Poly (lactic-co-glycolic acid)
PLLA: Poly (L-lactic acid)
RANK: Receptor activator of nuclear factor kappa-Β;
SEM: Scanning electron microscope
TCP: Tricalcium phosphate
TEM: Transmission electron microscopy
TGF: Transforming growth factor
WH: Whitlockite
XRD: X-ray diffraction spectroscopy
